# Data analysis in medical education research: a multilevel perspective

**DOI:** 10.1007/s40037-015-0160-5

**Published:** 2015-01-22

**Authors:** Jimmie Leppink

**Affiliations:** Department of Educational Development and Research, School of Health Professions Education, Maastricht University, 6200 MD PO Box 616, Maastricht, The Netherlands

**Keywords:** Medical education, Group learning, Repeated measurements, Multilevel analysis

## Abstract

A substantial part of medical education research focuses on learning in teams (e.g., departments, problem-based learning groups) or centres (e.g., clinics, institutions) that are followed over time. Individual students or employees sharing the same team or centre tend to be more similar in learning than students or employees from different teams or centres. In other words, when students or employees are nested within teams or centres, there is a within-team or within-centre correlation that should be taken into account in the analysis of data obtained from individuals in these teams or centres. Further, when individuals are measured several times on the same performance (or other) variable, these repeated measurements tend to be correlated, that is: we are dealing with an intra-individual correlation that should be taken into account when analyzing data obtained from these individuals. In such a study context, many researchers resort to methods that cannot account for intra-team and/or intra-individual correlation and this may result in incorrect conclusions with regard to effects and relations of interest. This comparison paper presents the benefits which result from adopting a proper multilevel perspective on the conceptualization and estimation of effects and relations of interest.

## Introduction

A substantial part of medical education research focuses on learning in teams (e.g., departments, problem-based learning groups) or centres (e.g., clinics, institutions) that are followed over time. Individual students or employees sharing the same team or centre tend to be more similar in learning than students or employees from different teams or centres [1]. In other words, when students or employees are nested within teams or centres, there is an intra-team or intra-centre correlation that should be taken into account in the analysis of data obtained from individuals in these teams or centres. Further, when individuals are measured several times on the same performance (or other) variable, these repeated measurements tend to be correlated, that is: we are dealing with an intra-individual correlation that should be taken into account when analyzing data obtained from these individuals [2–3]. This paper presents the benefits that result from adopting a proper multilevel perspective on the conceptualization and estimation in such a study context, a context that is quite common in medical education research. Many researchers still resort to methods that cannot account for intra-team and/or intra-individual correlation and this may result in incorrect conclusions with regard to effects and relations of interest.

### Context

Suppose, a researcher is interested in the effect of two types of group learning on test performance right after a course (i.e., immediate test performance) and one month later (i.e., delayed test performance), and decides to conduct a randomized experiment. The advantage of randomized experiments is that they allow for cause-effect inference much more than quasi-experimental and other types of studies.

**Fig. 1:**
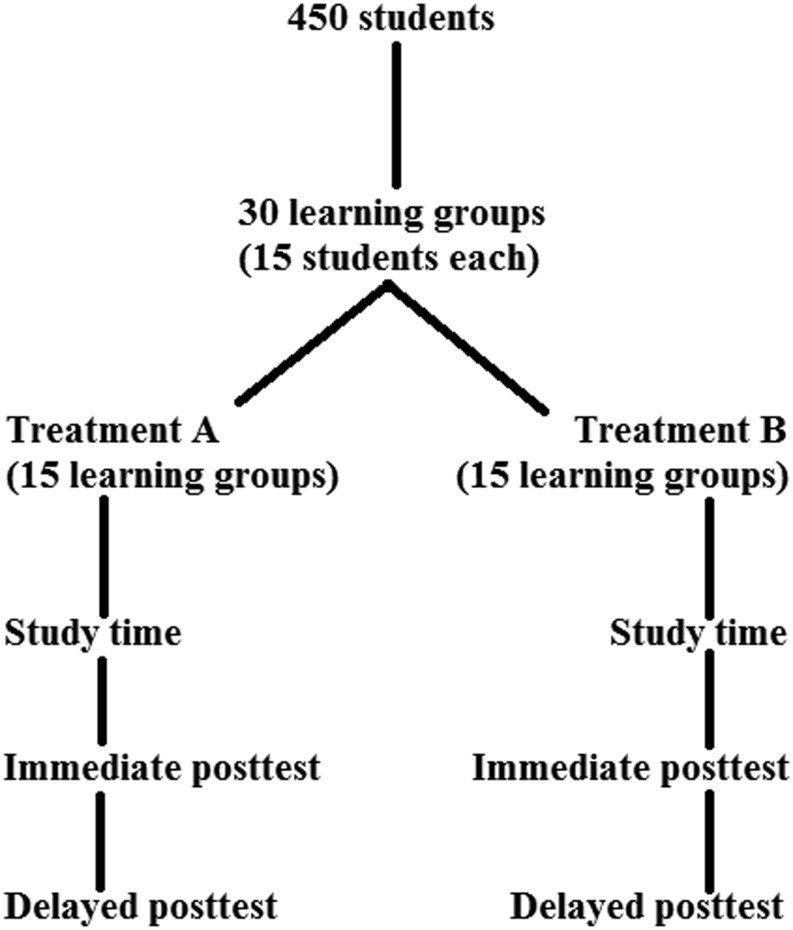
Study design used as example in this paper

The researcher decides to randomly allocate 450 students to 30 learning groups in such a way that every learning group comprises 15 students. Next, the learning groups are allocated randomly to either treatment A (control condition) or B (experimental treatment condition). The 15 A-groups study a medical problem by means of traditional cooperative learning, while the 15 B-groups study the same medical problem—and for the same interval of time as in the control group—but by means of a newly developed and more structured type of cooperative learning. The immediate test is administered directly after treatment. In the month after the test, students do not receive any additional treatment; they resume their usual study activities. At the end of the month, a delayed test is administered. Figure 1 visualizes the study design described.

This type of study design is also known as *split-plot design*. This term stems from agricultural experiments in which split plots of land received different treatments and were monitored or measured across time [4]. Likewise, in quite a number of educational and psychological experiments, students or other subjects are randomly allocated to different treatment conditions and are measured two or more times on the same performance or other variable [5]. In this example, we are dealing with such a design and we are confronted with one additional feature: treatment is not administered at the level of the individual student (as is the case in many psychological experiments) but at the level of learning groups in which the students take part (as is the case in more and more educational experiments). The following two hypotheses are to be tested:

- Hypothesis 1 (**H1**), *immediate treatment effect*: students who undergo treatment B perform better than their peers who undergo treatment A, on both immediate and delayed test; and
- Hypothesis 2 (**H2**), *treatment-by-time interaction effect*: students who undergo treatment A will lose more knowledge in the month following the immediate test than students who undergo treatment B.

In this context, we can distinguish between *fixed effects* and *random effects*. The purpose of a study like this is to generalize the findings to a larger population of (possible) students, and we assume that the students in our study form a random sample from a population that has a particular and preferably Normal (i.e., bell-shaped) distribution. In other words, we treat ‘student’ as a random effect. Treatment, however, is a fixed effect; we are interested in the specific comparison of treatments A and B, and we do not consider these two treatments to be a random sample of possible treatments to which we generalize. Likewise, in this context time is treated as a fixed effect; we are interested in differences in performance between two fixed time points, and we do not consider these two time points a random sample of possible time points to which we generalize. Finally, learning group (i.e., 15 students each) is treated as a random effect for the same reason as we treat student as a random effect; we consider the learning groups in our study a random sample from a population of (possible) learning groups that has a particular and preferably Normal (i.e., bell-shaped) distribution.

### Approach

In fact, we can distinguish three hierarchical levels in this problem: learning group (level 3: *k*), students nested within learning groups (level 2: *j*), and repeated measurements from the same students (level 1: *i*). Three common approaches to this type of research problems are:

- Single-level fixed-effects or *ordinary least squares* (OLS) regression in which the three hierarchical levels are treated as one single level and in which random effects of student and learning group are ignored;
- Split-plot *analysis of variance* (ANOVA) or two-level mixed-effects regression in which the learning group level (i.e., *k*) and thus random effect of learning group is ignored; and
- Three-level mixed-effects regression, which takes into account the full three-level hierarchical structure of the data.

We speak of *mixed-effects* analysis if our analysis involves at least one fixed and at least one random effect. Since all random effects are ignored in OLS regression, we call OLS regression fixed-effects analysis. The two-level approach includes the random effect of student, and the three-level approach includes the random effects of both student and learning group.

This paper compares the aforementioned three approaches, using this study context as example. For simplicity, this example uses equally large learning groups, an equal number of learning groups per treatment condition, and all students perform both the immediate and delayed test. We then speak of a ‘balanced design’. As a consequence of this design, all three approaches yield the same point estimates with regard to average treatment condition differences on immediate and delayed test. For unbalanced data, both OLS and split-plot ANOVA are likely to yield biased point estimates [2]. Further, it is demonstrated in this paper that, even for balanced data, OLS and split-plot ANOVA are biased in different ways with regard to variances and standard errors.

In the design at hand, two repeated measurements (level-1: *i*) are taken by 450 students (level-2: *j*) who are nested within 30 learning groups (level-3: *k*) that comprise 15 students each. The learning group level residual on the immediate test is a random, allowed-to-vary departure from the overall mean of the fixed-effect treatment condition on the immediate test, the student level residual on the immediate test is an allowed-to-vary random departure from the learning group level departure on the immediate test, and the change from immediate to delayed test is student-dependent.

## Method

For educational purposes, to allow for a good comparison of the three methods, data from this design were simulated in SPSS v19; a detailed overview of the simulation procedure is available from the author.

The advantage of a simulation study is that the outcomes of the study are known and as such it allows for a comparison of strengths and weaknesses of various methods of analysis, here: OLS regression, split-plot ANOVA, and three-level mixed-effects regression. All analyses were performed in MLwiN v2.10, a programme designed for multilevel analysis and suitable for a context like this, using for estimation fully informed maximum likelihood (FIML) for the fixed effects and restricted maximum likelihood (REML) for the random effects (in the two-level and three-level model) [3]. There is quite a variety of programmes that enable multilevel analysis (e.g., SAS, SPSS, STATA, HLM, SYSTAT, HLM, MLwiN, Mplus, R), and which programme is recommended depends on study design, types of variables under consideration, sample size, and a few other factors [6–9]. Since SPSS is a much more commonly used programme among medical education researchers than MLwiN, some instructions on how to do multilevel analysis in SPSS are provided in the Appendix.

### Between-subject effects

In OLS regression, all test outcomes from all students are assumed to be independent and identically distributed (i. i. d.). The research problem introduced previously is represented in the following equation:

$${{y}_{i}}={{b}_{0}}+{{b}_{1}}*\text{treatment}\_{{1}_{i}}+{{b}_{2}}*\text{time}\_{{1}_{i}}+{{b}_{3}}*\text{treatment}\_1*\text{time}\_{{1}_{i}}+{{e}_{i}},$$

where

*y*_*i*_ = test score *y* observed at data point *i*;

*b*_0_ = the average immediate test score in the control condition (A);

*b*_1_ = the difference between treatment conditions in average immediate test score;

*b*_2_ = the average change from immediate to delayed test in the control condition (A);

*b*_3_ = the difference between treatment conditions in average change from immediate to delayed test

(treatment-by-time interaction); and

*e*_*i*_ = the residual or random deviation from the fixed prediction.

The student-by-time interaction is ignored; all repeated measurements are assumed to be independent observations, as if they were 900 randomly sampled students attending one and the same lecture once in time. Due to the balanced design, the fixed point estimates *b*_0_ (44.076), *b*_1_ (11.089), *b*_2_ (− 4.036), and *b*_3_ (2.867) in OLS regression are the same as the fixed point estimates in the two-level and three-level model presented later on. However, the standard errors and residuals are different in each model.

### Within-subject effects

Repeated measurements data enable the researcher to separate within-subject variance from between-subject variance, and both types of variance are important in medical education research. Two types of correlation resonate in repeated measurements data: data sampling is hierarchical in that repeated measurements are taken from the same subjects (here: students), and educational measurement largely results from observation or self-reporting which creates serial correlation. Both types of correlation should be modelled appropriately. If students are measured more than twice, serial correlation observes special attention, for there are different types of serial correlation [2–3].

In the case of a balanced design with two repeated measurements, quite some researchers opt for split-plot ANOVA. In fact, this is a two-level mixed-effects model, in which student is the upper level (*j*) and the repeated measurements are correctly treated as observations from the same students. The within-subject (i.e., student) variance is separated from the between-subject variance, which is something that does not happen in OLS regression. The students participating in the study are assumed to form a random sample from a population that follows a particular (ideally: Normal) distribution. The OLS regression equation can be adjusted to derive the regression equation for the current model:

$${{y}_{ij}}={{b}_{0j}}+{{b}_{1}}*\text{treatment}\_{{1}_{j}}+{{b}_{2}}*\text{time}\_{{1}_{ij}}+{{b}_{3}}*\text{treatment}\_1*\text{time}\_{{1}_{ij}}+{{e}_{ij}},$$

where

*y*_*ij*_ = test score *y* observed for student *j* at repeated measurement *i*;

*b*_0*j*_ = the average immediate test score of person *j* in the control condition (A); and

*e*_*ij*_ = the residual or random deviation of student *j* at repeated measurement *i* from the fixed prediction.

In the equation above

$${{b}_{0j}}={{b}_{0}}+{{u}_{0j}},$$

and *u*_0*j*_ is the student-specific deviation in immediate test score with regard to *b*_0_. Note that *u*_0*j*_ and *b*_0*j*_ are random (not fixed) effects, hence the denomination mixed-effects model. In the standard error of a between-subject effect such as the experimental treatment effect, both within-subject and between-subject variance are present and cannot be separated. In the standard error of a within-subject effect such as change in test score over time the within-subject variance can and should be separated from the between-subject variance. It is for that reason that, as becomes clear in the results section later on, OLS regression yields a larger standard error for *b*_2_ and *b*_3_ than split-plot ANOVA.

### Learning groups

The learning group level is not taken into account in either OLS regression or in split-plot ANOVA. While the within-student between-measurement correlation is accounted for in split-plot ANOVA, the within-group between-subject correlation is not, and in OLS regression both types of correlation are ignored. Some researchers attempt to solve this by aggregating the data to the level of learning group. An average test score is then computed per repeated measurement for every learning group, and split-plot ANOVA is performed on the aggregated data. In this approach, the student level is wiped out of the analysis. Given that we (and researchers in medical education in a broader context) are also interested in the development of individual students, and effects on the individual student level can be different from effects on the learning group level, reducing the individual students’ data to their learning group average is not a feasible approach in medical education research.

A second group of researchers attempts to take the learning group level into account by including it as fixed effects in either an OLS regression model, or in a split-plot ANOVA. Either way, the fixed learning group approach is problematic for a number of reasons. First of all, in OLS regression and split-plot ANOVA, as discussed previously, the fixed effect of the model consumes four degrees of freedom (one for each of *b*_0_, *b*_1_, *b*_2_, and *b*_3_). When treating group as fixed factor, one needs dummy variables for the various learning groups and dummy variables for the learning group-by-time interaction. As a result, the fixed part requires 60 instead of four degrees of freedom (imagine the consequences if a study includes 300 learning groups). This affects standard errors greatly and results in highly inaccurate estimation. Moreover, no single parameter in the model addresses the treatment effect. Since each treatment condition comprises 15 groups, we cannot include both treatment and group as fixed effects in the model. Although in a balanced design we can compute the average learning group score for each treatment condition and this will indirectly lead to the treatment condition differences in average test score as obtained via the OLS regression or split-plot ANOVA model discussed previously, the learning group-specific standard errors in the model cannot be easily translated into one standard error for the treatment effect. The same problem is present in the analysis of the time effect and the treatment-by-time interaction effect. Finally, including learning group as fixed effect in the model disables generalization to other learning groups. One is generally interested in the effects of treatments A and B in *any* learning group that could apply one of these treatments. The 30 learning groups should therefore be considered as random learning groups; the first 15 learning groups form a random sample from a population of learning groups in which treatment A is applied, and the other 15 learning groups form a random sample from a population of learning groups in which treatment B is applied.

### Three levels

Multilevel analysis can take the hierarchical structure of the data into account in a way that none of the previously discussed approaches does, and enables correct analysis at each of the three levels: learning group (*k*), student (*j*), and repeated measurement (*i*). The appropriate multilevel model is a three-level mixed-effects model. In this model, the average immediate test score in the control condition, the difference in average immediate test score between the treatment conditions, the average change from immediate to delayed test in the control condition, and the difference in average change from immediate to delayed test between treatment conditions together form the fixed part. The random part can be fully explained by a combination of random intercept variance at the learning group level (*k*), and random intercept variance and random slope variance (and their covariance) at the student level (*i*), meaning that the residual on the lowest level—the level of the repeated measurements (*i*)—is equal to zero (and does not consume any degree of freedom). Consequently, the model consumes eight degrees of freedom, of which four for the fixed part and four for the random part (including one for the covariance between random intercept and random slope on the level of student). The full model is as follows:

$${{y}_{ijk}}={{b}_{0jk}}+{{b}_{1}}*\text{treatment}\_{{1}_{k}}+{{b}_{2j}}*\text{time}\_{{1}_{ijk}}+{{b}_{3}}*\text{treatment}\_1*\text{time}\_{{1}_{ijk}}\,+\,{{e}_{ijk}},$$

and given that *e*_*ijk*_, the residual on the level of repeated measurement i from student j in learning group k is equal to zero, the model can be reduced to

$${{y}_{ijk}}={{b}_{0jk}}+{{b}_{1}}*\text{treatment}\_{{1}_{k}}+{{b}_{2j}}*\text{time}\_{{1}_{ijk}}+{{b}_{3}}*\text{treatment}\_1*\text{time}\_{{1}_{ijk}},$$

where

*y*_*ijk*_ = the test score from student *j* from learning group k on repeated measurement *i*;

*b*_0*jk*_ = the immediate test score of student *j* from learning group *k* in the control condition (A);

*b*_1_ = the difference in average immediate test score between the treatment conditions;

*b*_2*j*_ = the change from immediate to delayed test for student *j* in the control condition (A); and

*b*_3_ = the difference in average change from immediate to delayed test between treatment conditions.

For *b*_0*jk*_ holds

$${{b}_{0jk}}={{b}_{0}}+{{v}_{0k}}+{{u}_{0jk}},$$

where

*b*_0_ = the average immediate test score in the control condition (A);

*v*_0*k*_ = the learning group-specific deviation in average immediate test score with regard to *b*_0_; and

*u*_0*jk*_ = the student-specific deviation in immediate test score with regard to *v*_0k_.

For *b*_2*j*_ holds

$${{b}_{2j}}={{b}_{2}}+{{u}_{2jk}},$$

where

*b*_2_ = the average change from immediate to delayed test in the control condition (A); and

*u*_2*jk*_ = the deviation in change from immediate to delayed test for student *j* in learning group *k*

with regard to *b*_2_.

Thus, the model can also be written as

$${{y}_{ijk}}=\left( {{b}_{0}}+{{v}_{0k}}+{{u}_{0jk}} \right)+{{b}_{1}}*\text{treatment}\_{{1}_{k}}+\left( {{b}_{2}}+{{u}_{2jk}} \right)*\text{time}\_{{1}_{ijk}}+{{b}_{3}}*\text{treatment}\_1*\text{time}\_{{1}_{ijk}}.$$

For student *j* in treatment condition A the immediate test score is (*b*_0_ + *v*_0*k*_ + *u*_0*jk*_), while for student *j* in treatment condition B the immediate test score is (*b*_0_ + *v*_0*k*_ + *u*_0*jk*_) + *b*_1_. For student *j* in treatment condition A the change from immediate to delayed test is (*b*_2_ + *u*_2*jk*_), whereas for student *j* in treatment condition B the change is (*b*_2_ + *u*_2*jk*_) + *b*_3_. The level-3 (*k*) residuals for the various learning groups *v*_0*k*_ are assumed to form a random sample from a normally distributed population with mean zero and variance $\sigma _{v0}^{2}$:

$${{\text{v}}_{0k}}\tilde{\ }\text{N}\left( 0,\sigma _{v0}^{2} \right).$$

The level-2 (j) residuals for the students nested within learning groups (*k*), *u*_0*jk*_ and *u*_2*jk*_, are also assumed to form random samples from normally distributed populations with mean zero and variance $\Omega u$:

$$\left[ \begin{aligned}& {{\text{u}}_{\text{0}jk}} \\& {{\text{u}}_{\text{2}jk}} \\\end{aligned} \right]\sim \text{N}\left( 0,{{\Omega }_{u}} \right).$$

## Results

Table 1 presents standard errors (*SE*) for each of *b*_0_ (44.076), *b*_1_ (11.089), *b*_2_ (− 4.036), and *b*_3_ (2.867), as well as random intercept variance at the learning group level (*k*), random intercept variance and random slope variance and their covariance at the student level (*j*), and the lowest-level residual (*e*) and associated *SE*s.

**Table 1:** Standard errors (*SE*) for each of *b*_0_ (44.076), *b*_1_ (11.089), *b*_2_ (− 4.036), and *b*_3_ (2.867), as well as random intercept variance at the learning group level (*k*), random intercept variance and random slope variance and their covariance at the student level (*j*), and the lowest-level residual (*e*) and associated *SE*s (between parentheses)

Model	OLS regression (single level)	Split-plot ANOVA (two levels)	Three-level mixed-effects
*SE*(*b*_0_)	1.481^a^	1.481^a^	5.344
*SE*(*b*_1_)	2.095^a^	2.095^a^	7.557
*SE*(*b*_2_)	2.095^b^	0.267	0.267
*SE*(*b*_3_)	2.962^b^	0.378	0.378
*s* ^2^(*v*_0_ *k*)	–	–	422.301 (110.551)
*s* ^2^(*u*_0*jk*_) (*SE*)	–	485.599 (32.498)^c^	90.020 (6.212)
*s* ^2^(*u*_2*jk*_) (*SE*)	–	–	16.048 (1.070)
*cov*(*u*_0*jk*_, *u*_2*jk*_) (*SE*)	–	–	0.980 (1.854)
*e* (*SE*)	493.624 (23.167)	8.025 (0.533)^d^	0.000 (0.000)

^a^underestimation of *SE* due to overestimation of degrees of freedom

^b^overestimation of *SE*, since within-subject variance is not separated from between-subject variance

^c^ *u*_0*j*_ for this model, since *k* is ignored here

^d^this is the difference between 493.624 and 485.599; it is the variance assumed for both treatment conditions

OLS heavily overestimates the standard errors for *b*_2_ and *b*_3_, effects in which within-subject variance plays a role. The within-subject variance is separated from the between-subject variance in split-plot ANOVA and the three-level model, and as a consequence, the standard errors for *b*_2_ and *b*_3_ are much smaller than according to OLS. Ignoring the learning group level does not affect the standard errors for *b*_2_ and *b*_3_ in the split-plot ANOVA. This is because within-subject effects have different variances and degrees of freedom than between-subject effects, and ignoring the learning group level only affects the degrees of freedom of between-subject effects. This also explains why the standard error for *b*_1_ is underestimated in both OLS and split-plot ANOVA.

## Conclusion

The advent of the personal computer with more and more computational power resulted in an increased use of multilevel models [10]. Nonetheless, many still use OLS and related ANOVA approaches for multilevel data because they are used to it. For instance, in experimental psychology there is a longstanding tradition of using ANOVA models, and OLS is typically (over)used in much of health research. Many researchers continue using ANOVA or OLS because they ‘have always done it like that’ and think that ‘a more complex analysis does not make much difference anyway.’ Indeed, there are situations when a more complex analysis does not make much difference. That is, when little to no interaction between students within groups results in very little within-group dependency, taking into account the group level may not result in substantially different outcomes with regard to the effects or relations of interest. However, this is not the norm, and even smaller within-group dependency should make researchers examine what adding the group level changes in outcomes [1].

### The beast of aggregation

In any case, aggregating student-level data to some group average does not resolve the phenomenon of within-group dependency and is rarely if ever a good approach to deal with such dependency. This also holds for situations where for instance groups of students trained by the same clinical teacher have to rate teaching skills or other characteristics of that teacher. While a common argument to ‘justify’ aggregation is that such ratings aim at ‘evaluating the performance of an individual clinical teacher at the workplace’ [11], students rarely provide exactly the same ratings, some clinical teachers may receive more ratings than others, and some clinical teachers may receive more varied ratings than others. All this information is lost when aggregating students’ data to one single average score per teacher, and this can have major influences on effects and relations of interest, including negative correlations being artificially changed into positive ones and vice versa [1]. Therefore, do not wipe out the student level through aggregation.

A similar reasoning holds for repeated measurements. Recently, a series of well-designed randomized experiments provided evidence for the statement that in studies where learners have to perform a series of tasks, it is better to measure a characteristic of interest—for instance mental effort—after each task (i.e., repeatedly) than once retrospectively [12]. This is an excellent statement, for repeated measurements data enable the researcher to separate within-subject variance from between-subject variance, and both types of variance are important in (medical) education research. However, if we aggregate these repeated measurement data to one average score to then correlate that average score to some other (perhaps also aggregated) variable, we fall in the same trap of aggregation and can face potential serious distortions of effects and relations of interest [1].

### N students being measured k times does not equate N times k independent observations

Ignoring intra-individual correlation as is done in OLS is unfortunately still quite common in (medical) education research, including in high-quality research published in respectable journals. For instance, in a recent study, six medical residents who individually interpreted eight electrocardiograms (ECGs) were treated as a ‘sample size of 48’ (i.e., six times eight) on which something comparable to OLS regression was performed [13]. This is like seeing 48 residents who independently rated one single ECG. In the latter case, assuming 48 independent observations could be realistic. In the current context, however, there is a within-resident between ECG/interpretation correlation that reduces the number of independent observations to somewhere between the number of residents (i.e., six) and the total number of data points (i.e., six times eight).

A slightly different yet similar approach is chosen when researchers perform separate ANOVAs to test for group differences at each time point instead of accounting for the fact that at least considerable proportions of students have taken multiple tests and that students taking the test at some point may have been nested within learning groups [14]. Even if testing for group differences at a specific time point is legitimate from an interest in group differences at that very point in time, ignoring group nesting and the intra-group correlation that goes with it tends to result in an overestimation of the number of independent observations at that time point and this may exaggerate to some extent the statistical significance of a group difference at that time point.

One thing should be added, before turning to the next section. The papers used as examples of studies in which a multilevel approach should or could have been used [11–14] were not chosen because of a lack of quality. On the contrary, each of the papers discussed presents high-quality research published in a respectable journal. However, it is well possible that adopting a multilevel analysis approach would have resulted in somewhat different conclusions with regard to some effect(s) or relation(s) of interest. These papers illustrate that even in the case of a well-designed study, different approaches to analysis do exist and it is worth thinking carefully about which analysis approach accounts for your study design and data to an optimal extent. There is a metaphorical bridge between research questions, study design, and data analysis; the study design is supposed to logically follow from your research questions and should be reflected in the data analysis stage. Even if both optimal and suboptimal approaches of analysis result in a statistically significant *p*-value for a particular effect or relation of interest, statistics is not in the first place about *p*-values; it is rather about the mathematical modelling of empirical phenomena. For instance, it is possible that in a particular context OLS regression yields a statistically significant positive correlation between two variables where appropriate multilevel regression yields a statistically significant negative correlation between the same variables.

In the following section, some benefits of multilevel analysis to the aforementioned approaches are discussed.

### Some benefits of multilevel analysis

Compared with the approaches discussed previously, appropriate multilevel analysis has a number of benefits on the conceptualization and estimation in a study context as discussed in this paper.

Firstly, multilevel analysis stimulates the researcher to conceptualize and specify the various levels in the research design, so that the variance at each level can be modelled and estimated correctly. Multilevel analysis is the only approach that enables the researcher to conceptualize the hierarchical structure of the data and specify the hierarchical levels in the data correctly and completely. In split-plot ANOVA, the given three-level mixed-effects structure is reduced to a two-level mixed-effects structure, and in OLS regression it is reduced to a one-level fixed-effects structure. The fixed learning group approach results in non-interpretable models that consume many degrees of freedom and the estimates cannot be used to generalize to other learning groups in which treatment A or B is or could be applied.

Secondly, whether one is interested in estimating between-subject or within-subject effects or a combination of these two types, multilevel modelling enables the researcher to model and estimate these effects appropriately. OLS regression results in underestimated standard errors of between-subject (here: treatment) effects and overestimated standard errors of within-subject (here: time) effects and split-plot interaction (here: treatment-by-time) effects, whereas the split-plot ANOVA approach results in underestimated standard errors of between-subject effects. While standard errors of within-subject and split-plot interaction effects are inflated when intra-student correlation is ignored, ignoring intra-group correlation induces a downward bias in standard errors of between-subject effects. Standard errors affect outcomes of statistical significance tests and the width of confidence intervals around the point estimates (i.e., the latter are used for interval estimation). Underestimated standard errors result in a larger Type I error probability in hypothesis testing (i.e., incorrect rejecting of a true null hypothesis) and too narrow confidence intervals for an effect or relation of interest. Overestimated standard errors result in larger Type II error probability in hypothesis testing (i.e., failing to reject an untrue null hypothesis) and too wide confidence intervals for an effect or relation of interest. Either of the two can result in incorrect conclusions with regard to (treatment) effects and relations of interest, and this is not a good thing if we decide to use the outcomes of our analyses for curriculum and policy making in (medical) education.

Thirdly, in all approaches discussed in this paper except for the three-level model, different types of homogeneity assumptions are made. OLS regression assumes homogeneity of variance across combinations of treatment condition and repeated measurements (which is what ‘identically’ in i. i. d. refers to), meaning for the design discussed in this paper that the variance in immediate test score is equal for both treatment conditions and equal across repeated measurements. In split-plot ANOVA, homogeneity of the covariance matrix for both treatment conditions is assumed. Both types of homogeneity assumptions are frequently violated, and not taking these violations into account can lead to serious bias in standard errors and interval estimation. Further, in designs in which students undergo more than two repeated measurements, different serial correlation structures can arise. Multilevel analysis enables the researcher to model heterogeneity of variances and potential serial correlation easily. In the study discussed in this paper, difference in variance between immediate and delayed test score and between treatment conditions is modelled by the inclusion of the random intercepts and random slope. The random part is modelled completely, whereas in all other approaches discussed in this paper a considerable proportion of the random part remains unexplained.

Fourthly, especially in non-experimental studies, unbalanced designs (e.g., learning groups of varying size, missing data) are to be expected, and then split-plot ANOVA and OLS regression yield biased point estimates. However, even in experimental studies, dropout can occur, and then multilevel analysis generally provides a less biased and more efficient approach than split-plot ANOVA, OLS regression or similar approaches [15].

### A note on the number of levels

The need for a three-level mixed-effects model in this paper arises from the presence of both intra-individual correlation and intra-group correlation. The intra-individual correlation results from the same individuals being measured twice, while the intra-group correlation is due to the fact that individuals were learning in groups of 15. Had only one test been administered (instead of two), there would have been no repeated measurements and thus no intra-individual correlation to be taken into account in the analysis. In that case, a two-level model with group (level-2: *j*) and individual student (level-1: *i*) would have been appropriate. This may hold even if group size is as small as two students [16].

Likewise, had treatment not been administered at the level of groups of collaborating individuals but at the level of the individual, there would have been no intra-group correlation to be taken into account in the analysis. If then students were still measured twice (i.e., immediate and delayed test), a two-level model with individual student (level-2: *j*) and measurement occasion (level-1: *i*) would have been appropriate [15]. OLS could have been appropriate had treatment been administered at the level of the individual and only one test was administered (i.e., no repeated measurements).

Many medical education research questions focus on learning in teams or clinics and/or learning over time. In this context, medical education research could profit from the benefits of multilevel analysis more than it has done until now. This paper demonstrates what can happen when resorting to a frequently used but suboptimal method of analysis and provides an approach that can be used by other researchers dealing with this kind of data.

## Essentials

- When individuals are nested within teams, there is an intra-team correlation that should be taken into account in the analysis of data obtained from individuals in these teams or centres.
- The intra-individual correlation resulting from individuals being measured two or more times should be taken into account when analyzing data obtained from these individuals.
- Multilevel analysis enables the researcher to conceptualize the hierarchical structure of research data and appropriately account for intra-individual and/or intra-team correlation.
- Traditional regression and analysis of variance methods fall short in dealing with intra-individual and/or intra-team correlation and are therefore generally not recommended in such a context.
- Much of (medical) education research is about individuals nested within teams and/or individuals measured repeatedly; therefore, (medical) education research provides a natural context for multilevel analysis.

### Source(s) of support in the form of grants

None.

## Appendix

This appendix provides some instructions on how to do multilevel analysis in SPSS v19 and later. For a more detailed tutorial of multilevel analysis in SPSS, Chap. 20 in Andy Field’s Discovering Statistics Using IBM SPSS Statistics is recommended [17].

## Data entry

In studies in which you have individuals nested within learning groups or a similar kind of random groups, take care that your data file has as many rows as there are individuals and that you have two separate columns for group and individual code, respectively. This way, you communicate to SPSS that row X in the data file has all the data from individual *j* in learning group *k*.

In studies in which individuals have been measured repeatedly on the same performance or other variable, take care that the number of rows in your data file equals the number of individuals times the number of repeated measurements (i.e., *univariate* or *long* data file). This way, you communicate to SPSS that row X in the data file corresponds with individual *j* measured at time point *i*. This is different from what people trained in classical repeated measures ANOVA are used to, because there you typically have one row per individual and for the response variable measured repeatedly as many columns per row as there are repeated measurements (i.e., *multivariate* or *wide* data file).

Note that additionally you still need to use columns for the other variables of interest (e.g., treatment, gender, age, grade-point average).

## Restructuring and screening

It is also possible to save the data once in *univariate* and once in *multivariate* format. Although you can order relevant descriptive statistics and graphical output in both formats (in the multivariate format, you can use the *Split file* or *Select cases* function in the *Data* menu), many people are used to doing much of the descriptive and graphical work in the multivariate format. Further, it is relatively easy to restructure a multivariate data file into a univariate data file and vice versa, using the *Restructure* function in the *Data* menu. With the first option (i.e., *Restructure selected variables into cases*) you can restructure a multivariate data file into a univariate data file, and with the second option (i.e., *Restructure selected cases into variables*) you can restructure a univariate data file into a multivariate data file. When using the *Restructure* function, it is recommendable to save the file under a different name, so that both files remain available just in case you make a mistake in the restructuring process.

## Mixed models

The terms *multilevel model*, *mixed model*, *mixed-effects model*, and *hierarchical model* are used interchangeably for the same type of model, a model in which two or more (hence *multi*) hierarchically structured (hence *hierarchical*) levels are taken into account (as done in the two-level and three-level model in this paper) and some effects are fixed (e.g., treatment) while other effects are random (hence *mixed*).

It is therefore of little surprise that much of multilevel analysis in SPSS can be done in the *Analyze* menu through *Mixed Models*. The *Generalized Linear Mixed Models* option provides some options for multilevel analysis when dealing with categorical response variables or when dealing with quantitative response variables that have a specific non-Normal distribution. For the kind of study discussed in this paper, we can use the *Linear* option.

## Specify subjects and repeated

So we go to *Analyze*, *Mixed Models*, and then choose *Linear*. The screen that pops up, *Linear Mixed Models: Specify Subjects and Repeated*, asks us to specify subjects and/or repeated measurements variables.

The *Subjects* field is used to define if we have some group nesting going on in our study. If so, we first drag the variable that defines the random groups to the field and subsequently the variable that defines the individuals. Thus, in the example discussed in this paper, we would first drag the ‘learning group’ variable and then the ‘student’ variable to that field.

The *Repeated* field is used for defining repeated measurements variables. In the example discussed in this paper, that would be the ‘time’ variable. Had the students in our example study not been nested within learning groups, we would have needed to drag only the ‘students’ variable to the *Subjects* field and the ‘time’ variable would still be in the *Repeated* field.

At the end of the menu, you see *Repeated Covariance Type*. For the example study discussed in this paper, you could put that on *Scaled Identity*, because we model the random effects through random intercepts and random slopes later on. Elaborating on the meaning of some of the repeated covariance types listed in that menu would require an extensive explanation that could easily fill a full paper by itself [2–3].

## Dependent variable, factor(s), covariate(s)

After you click ‘continue’ in the first screen, a second screen—*Linear Mixed Models*—appears. On the left hand of the menu you see all variables, while on the right hand you see three fields: *Dependent Variable*, *Factor(s)*, and *Covariate(s)*.

The dependent or response variable is the variable we want to predict or compare across groups, in the current example ‘test score’. The Factor(s) field is useful if we are interested in, for instance, testing for differences in average test score between more than two groups (including post hoc tests involving pairwise comparisons). An easy way of analysis in our example is to use 0/1 coding for the two treatments and for the time variable and include these two variables in the Covariate(s) field. There is much more to say on coding [6] and Factor/Covariate difference [17], but elaborating on this would require a substantial extension of this paper.

## Fixed

In the ‘fixed’ submenu, you can specify the fixed part of the model. In our example study, that would be ‘treatment’, ‘time’, and ‘treatment *by* time’. This is because ‘treatment’ and ‘time’ are considered fixed effects in our example.

## Random

The ‘random’ submenu allows you to specify the random part of the model. On the downside of the submenu, you see under *Subjects* the variables listed that you previously (i.e., in the *Specify subjects and repeated* screen) dragged into the ‘Subjects’ field. When you drag, for instance, the ‘learning group’ variable to the *Combinations* and hit the *Include intercept* box, you specify the learning group-level random intercept. To also model a random intercept and/or slope for ‘student’, we need to click ‘next’ on the upper right of the submenu, there drag ‘student’ to *Combinations*, hit the *Include intercept* box for a student-level random intercept and drag the ‘time’ variable from the *Factors and Covariates* field to the *Model* field. Then click ‘continue’.

## Estimation

The default method chosen in the ‘estimation’ submenu is REML. This method is preferred when estimating and testing random effects; the second method, ML, is generally preferred when estimating and testing fixed effects [3]. For many studies in medical education where a two-level or three-level linear model could be applied, the other settings in the ‘estimation’ submenu can generally be left to the default options, only perhaps increasing *Maximum scoring steps* to 10 (instead of 1).

## Statistics

In the ‘statistics’ submenu, we generally check *Parameter estimates* and *Tests for covariance parameters*, and—depending on what our model looks like—also *Correlations of parameter estimates* and/or *Covariances of parameter estimates*, and *Covariances of random effects* and/or *Covariances of residuals*.

## Estimated Marginal Means and Save

The ‘EM Means’ (i.e., estimated marginal means) submenu is especially useful when dealing with factors that comprise more than two groups and want to obtain estimates of group means. Using the ‘Save’ submenu requires a more profound understanding of multilevel analysis and regression analysis in a broader perspective.

## Syntax

Once you have specified and ordered everything as intended, you can click ‘OK’ or better ‘Paste’. The great advantage of the ‘Paste’ function is that SPSS saves the model you want to run in a few lines of ‘syntax’ in a separate syntax file. You can save the syntax file separately and return to your analysis at a later point in time.

## Final note

Of course, much more can be said about multilevel analysis than has been done in this paper and appendix. However, various key references have been provided for readers who are interested in bringing it further [1–3, 6–10, 17].
